# Multidrug-resistant *Pseudomonas aeruginosa* infections: current status, challenges, and prospects of phage therapy

**DOI:** 10.3389/fmicb.2025.1723885

**Published:** 2026-01-21

**Authors:** Bo Yan, Can Yan, Yafang Ding, Siyi Cai, Yujin Wang, Elvis Agbo, Xianyun Xu, Kunhao Qin, Qiang Fu

**Affiliations:** 1 Affiliated Hospital of Jinggangshan University, Ji’an, China; 2 Jiangxi Province Key Laboratory of Organ Development and Epigenetics, Clinical Medical Research Center, Affiliated Hospital of Jinggangshan University, Medical Department of Jinggangshan University, Ji'an, China; 3 Department of Clinical Laboratory, Ji'an Central People's Hospital, Ji’an, China

**Keywords:** MDR-PA, infections, bacteriophage, therapy, antimicrobial resistance

## Abstract

The emergence of drug-resistant bacterial infections has profoundly impacted global public health. Key pathogens include multidrug-resistant *Pseudomonas aeruginosa* (MDR-PA), MDR *Acinetobacter baumannii*, and methicillin-resistant *Staphylococcus aureus*. Among these pathogens, MDR-PA carries numerous virulence factors that induce extensive tissue destruction. Its inherent ability to form biofilms promotes chronic infection persistence and multidrug resistance, leading to mortality rates up to 40%. Currently, antibiotics remain the mainstay for the treatment of MDR-PA infections. Nevertheless, the escalating prevalence of drug resistance has rendered conventional antibiotic regimens increasingly recalcitrant. Consequently, the imperative for innovative antimicrobial therapeutic modalities to combat *Pseudomonas aeruginosa* has intensified in the realm of public health. In this context, phage therapy, with its precise bactericidal activity and high host biosafety, has emerged as a compelling alternative. This review provides a comprehensive synthesis of recent advancements in phage therapy targeting MDR-PA, covering clinical applications, current therapeutic approaches, and emerging technological platforms. It further dissects the resistance mechanisms encountered during treatment and puts forward novel counterstrategies to address antimicrobial resistance challenges—including optimized phage-antibiotic synergy, phage genome engineering, and dynamic adaptive therapeutic frameworks—aimed at advancing clinical translation.

## 1 Introduction

*Pseudomonas aeruginosa* (PA) is a Gram-negative aerobic bacillus capable of surviving in water, on surfaces of all kinds, and on medical devices by means of binding factors such as flagella, pili, and biofilms. This characteristic renders infection with the bacterium a serious threat to patients with cystic fibrosis, cancers, severe burns, and immunocompromised systems and leads to a significant increase in mortality in these patients (Nanayakkara et al., 2021; Appaneal et al., 2022). Furthermore, Carbapenem-resistant PA is classified by the World Health Organization as a critical priority pathogen for research and development of new antibiotics (Miller and Arias, 2024; Weimann et al., 2024; Macesic et al., 2025). A recent study published in The Lancet indicated that PA, in conjunction with four other pathogens (*Staphylococcus aureus*, *Escherichia coli*, *Streptococcus pneumoniae*, and *Klebsiella pneumoniae*), accounts for 54.9% of bacterial-associated fatalities (Ikuta et al., 2022). This figure designates it as the second-leading cause of bacterial deaths on a global scale, thereby presenting a substantial challenge to healthcare systems globally.

The treatment of PA dates back to the 1940s, when penicillin offered an effective therapy. However, Alexander Fleming, the discoverer of penicillin, early warned that antibiotic misuse could drive resistance in pathogenic organisms (Gerberi, 2024). As he feared, reports of drug-resistant PA strains quickly came to light. The primary mechanisms of resistance include reduced permeability of the outer membrane, which restricts antibiotic entry; production of antibiotic-inactivating enzymes, which can inactivate antibiotics; biofilm formation, which provides a protective barrier for the bacteria; and expression of the active efflux system, which excretes antibiotics out of the bacterium. For instance, the upregulation of efflux pump genes substantially augments both the number and activity of these pumps, which efficiently expel intracellular antibiotics, thereby reducing the drug’s effective concentration and compromising its bactericidal effect (Vergalli et al., 2020). New antibiotics, including aminoglycosides, cephalosporins, and carbapenems, were developed in the subsequent decades. However, with the growing problem of antibiotic resistance, a single antibiotic regimen is no longer sufficient for PA infections. Consequently, since the 1970s, combination therapies, including *β*-lactams with aminoglycosides or quinolones, have been adopted to improve efficacy and slow resistance emergence (Choi et al., 2023). While this approach has led to a notable expansion in the range of clinical applications, it has also given rise to concerns regarding the potential evolution of bacterial cross-resistance and the emergence of a broader array of resistance mechanisms, such as tetracycline-inactivating enzymes (Blake et al., 2024).

Antibiotic resistance-related mortality has now ascended to the third-leading cause of death globally. According to the Centers for Disease Control and Prevention, there are more than 2.8 million antimicrobial-resistant infections and over 35,000 associated deaths each year in the United States (Flynn and Guarner, 2023). A significant contributor to this crisis is multidrug-resistant (MDR) PA, which is estimated to cause 32,600 cases and 2,700 deaths each year, with some strains exhibiting resistance to nearly all available antibiotics (Tenover et al., 2022). Concurrently, the Council of Canadian Academies’ assessment report forecasts that, in the absence of an efficacious solution to drug-resistant bacterial infections, the mortality rate from such infections will exceed that from cancer by 2050, resulting in 11 million new deaths (Pulingam et al., 2022). These realities and predictions underscore the pressing need to combat MDR PA resistance on a global scale. Thus, there is an urgent imperative to fortify research and development efforts for novel antimicrobial therapies and to rationalize the utilization of existing antibiotics in the “post-antibiotic era.” These measures are crucial to curbing the propagation of drug-resistant bacteria and their deleterious effects. A multitude of promising alternatives are currently being investigated, including antisense oligonucleotides, honey, antimicrobial peptides, microbiota, probiotics, metals, antimicrobial enzymes, and plant extracts (Abuelgasim et al., 2021; Aghamohammad and Rohani, 2023; Cesaro et al., 2023; MacNair et al., 2024). Among these, phages have resurged as non-antibiotic antimicrobial agents due to their ability to control bacterial infections with greater efficacy and their distinct bactericidal properties.

Phage therapy, a non-antibiotic therapeutic strategy, has demonstrated considerable promise in combating MDR pathogens, such as PA. Unlike conventional broad-spectrum antibiotics that act indiscriminately, phages specifically recognize and infect host bacteria, achieving precise bactericidal effects through their unique lytic cycle (Penadés et al., 2025). This process initiates with the highly efficient adsorption of receptor-binding proteins on the phage surface to specific receptors on the bacterial cell. Subsequently, the phage injects its genetic material into the host cytoplasm and hijacks the bacterial transcription and translation machinery to synthesize and assemble viral components. Towards the end of the lytic cycle, the phage-encoded endolysin, which is a hydrolase that specifically cleaves peptidoglycan, disrupts the cell wall, ultimately leading to osmotic lysis and the release of progeny virions (Nawaz et al., 2022; Khan et al., 2023; Liu et al., 2023). Furthermore, some phages produce polysaccharide depolymerases that degrade the bacterial extracellular polymeric substances, thereby potentiating the antimicrobial efficacy (Gliźniewicz et al., 2023; Guo et al., 2023). Collectively, this highly specific bactericidal mechanism not only effectively targets drug-resistant bacteria but also circumvents damage to the commensal flora, thus maintaining microbiological homeostasis.

To date, a substantial body of global preclinical research and Phase I/II clinical trials has provided preliminary evidence supporting the safety and efficacy of this therapeutic approach. This review comprehensively examines advances in phage therapy against drug-resistant PA infections, encompassing research and clinical applications. It also discusses key challenges and emerging technologies, and critically evaluates the translational value of phage therapy across diverse contexts and advance its integration into clinical practice.

## 2 Preclinical evidence for phage therapy against PA

A rigorous, stepwise preclinical evaluation, spanning from *in vitro* assays to *in vivo* animal infection models, is fundamental to validating the therapeutic potential of phage therapy against MDR PA. At the in vitro level, investigations have focused on quantifying direct bacteriolytic activity, eradicating established biofilms, and identifying synergistic interactions with conventional antibiotics. *In vivo*, studies utilizing various animal models have further delineated treatment efficacy under physiologically relevant conditions, optimized delivery routes and dosing, and concurrently evaluated the biosafety profile of phage formulations. This section synthesizes key findings from these studies, which collectively aim to establish robust proof-of-concept, optimize treatment regimens, and elucidate the therapeutic profile of phage therapy prior to clinical translation.

### 2.1 *In vitro* experiments of phage therapy for the treatment of PA infection

Considering the escalating prevalence of MDR PA infections, researchers have initiated investigations on the potential use of phage therapy. Bernabéu-Gimeno et al. proposed a systematic classification of therapeutic strategies based on phage species, distinguishing between monophage therapy and multiphage therapy (Bernabéu-Gimeno et al., 2024). This categorization framework provides a theoretical basis for assessing the effectiveness of different treatment modalities. The potential of a combined multiphage therapeutic strategy was validated by the experiments of Alves’ team (Alves et al., 2016), which demonstrated that the phage mixture cleared more than 95% of the biofilm within 4 h under static conditions (multiplicity of infection [MOI] = 10) and that the mixture retained a significant inhibitory effect on biofilm biomass and structure after 48 h in a dynamic flow model of this experiment. It is worth noting that this multiphage synergistic effect may curtail the risk of PA developing resistance mutations by targeting multiple host receptors. This contrasts with the challenges posed by single-phage therapies. The limitations of single-phage therapy are thoroughly delineated in the study by Li et al., who identified three phage-resistant mutant strains: phipa2-R, phipa4-R, and phipa10-R (Li et al., 2022). Although the strains’ motility was significantly reduced (migration distances were reduced to 47, 80, and 30% of the wild strains, respectively), they showed a differential evolution in biofilm-forming capacity: The biofilm generation of phipa2-R and phipa4-R was reduced to 0.7 and 0.5, but the biofilm production of phipa10-R was paradoxically elevated to 3.0 (1.0 in the wild strain). This paradoxical phenomenon suggests that the host-adaptive trade-offs triggered by phage resistance are strain specific. Biofilm regulatory mechanisms may be compensatorily altered through different molecular pathways. The studies of Bernabéu-Gimeno’s and Alves’ teams provide evidence that multiphage therapies are effective in inhibiting biofilms by targeting multiple receptor pathways. However, the discovery of phipa10-R phenotypic heterogeneity by Li et al. indicates that a single phage selection pressure may unexpectedly enhance the pathogenicity profile of specific mutant strains. These findings illuminate the intricacies of phage–host interactions from the perspective of evolutionary dynamics and offer two insights that could inform the optimization of therapeutic strategies. On the one hand, phage combinations that interfere with virulence factors (e.g., biofilm synthesis-associated receptors) should be prioritized. On the other hand, the adaptive compensatory effects caused by resistance mutations should be avoided through multi-target synergistic effects.

Recent studies have demonstrated that the combination of phages and antibiotics can significantly enhance the antimicrobial therapeutic effect through a multidimensional mechanism. The experimental data demonstrate that phages can effectively reduce the minimum inhibitory concentration (MIC) of antibiotics (Wang et al., 2024; Pirlar et al., 2025). Meanwhile, sublethal concentrations of antibiotics specifically activate bacterial biosynthetic pathways, promoting the accumulation of biomass (e.g., polysaccharides and proteins) (Li et al., 2021; Hatfull et al., 2022). This process creates optimized conditions that enhance phage proliferation. Therefore, when these two elements are utilized together, they can elicit substantial synergistic bactericidal effects. This distinctive biological response mechanism provides a theoretical foundation for the development of combination therapy programs that utilize sublethal doses of antibiotics. When evaluated from the standpoint of evolutionary dynamics, this combination therapy strategy establishes evolutionary constraints through two mechanisms of action. Phages disrupt the cell wall structure and biofilm integrity through lysis, while antibiotics interfere with bacterial metabolic processes or protein synthesis pathways (Chen et al., 2022; Abedon, 2023). Two distinct targets of action generate a synergistic evolutionary pressure, thereby hindering bacteria from developing adaptive resistance to both classes of drugs concurrently. It has been demonstrated that this strategy not only enhances the immediate antimicrobial effect but also significantly delays the emergence of drug-resistant mutant strains, which is of strategic value for solving the global antibiotic resistance crisis.

It should be emphasized that chronomodulation is a key therapeutic strategy in phage-antibiotic combination therapy. Recent studies demonstrate that optimizing the timing and sequence of administration significantly enhances the therapeutic efficacy of this combined approach while simultaneously counteracting the evolution of drug resistance. For instance, Torres-Barceló’s team discovered through dynamic modeling that delaying streptomycin administration until 12 h after phage treatment reduced bacterial density by nearly six orders of magnitude and sustained an inhibition of 60% while keeping the incidence of resistance below the threshold (Torres-Barceló et al., 2014). This finding stands in stark contrast to the accelerated bacterial resuscitation observed in the monotherapy group, thereby substantiating the notion that meticulous regulation of dosing timing can circumvent the constraints imposed by conventional therapeutic modalities. It is worth noting that this timing-dependent effect demonstrated no significant correlation with antibiotic dosage, indicating that the temporal aspect of therapy may hold greater clinical significance compared to dosage optimization. Consequently, there is an urgent need to establish a multi-parameter assessment system in current research. This system should focus on three key areas: (1) quantifying the molecular basis of the synergistic effect of different antibiotic classes with phages, (2) resolving the influence of the biofilm microenvironment on the effect of combination therapy, and (3) developing a multidrug sequential dosing model based on evolutionary game theory. These research directions will promote the leapfrog development of phage-antibiotic combination therapy from experimental discovery to clinical translation.

### 2.2 Animal model of phage therapy for PA infection

A recent study by Cieślik’s team revealed the dose–effect relationship and immunomodulatory properties of phage treatment for PA infection through the synergistic validation of invertebrate and vertebrate models (Cieślik et al., 2021). In a PA-infected giant wax borer model, the administration of the phage was controlled chronologically (1 h between bacteria and phage), resulting in a typical MOI dependence of 72-h survival. Survival increased from 12.5 to 87.3% when the MOI was 0.1, and histopathological analysis revealed that epidermal integrity indices in the phage-treated group were significantly superior to those in the untreated group. Similarly, in a zebrafish cystic fibrosis model, a dual time-point intervention strategy (30 min + 7 h post-infection) with a tetravalent phage cocktail (2 × 10^7^ PFU) resulted in a significant increase in embryo survival. The findings of both experiments underscore the pivotal role of temporal parameters and phage therapy dosage in determining the treatment’s efficacy. The application of bacteriophages has been demonstrated to be a rapid and effective method for reducing bacterial infections. Therefore, precise timing and dosage of administration are crucial to ensure the success of phage therapy. A notable finding from the RNA sequencing analysis of the zebrafish model was the significant decrease in pro-inflammatory factors, including TNF-*α* and IL-1β. This observation suggests the potential for phages to regulate immune homeostasis during infection, thereby reducing the population of PA. The dual mechanistic action—combining targeted pathogen elimination with host immune modulation—confers exceptional therapeutic precision against multidrug-resistant infections, underscoring phage therapy’s translational value beyond conventional antimicrobial paradigms.

In a similar vein, phage therapy has exhibited dose-effect advantages and synergistic therapeutic potential in higher biological models of PA infection. Abdelghafar’s team developed a pharmacokinetic-pharmacodynamic (PK/PD) correlation model of intraperitoneal infection in mice, wherein a single phage injection reduced hepatic PA bacterial load by 2 log_10_, concomitantly with a substantial reduction in splenic levels of the inflammatory factor IL-6. Time series analysis demonstrated that the 24-h survival rate in the treated group reached 70.0%, which was significantly higher than that in the untreated group, which was 0% (Abdelghafar et al., 2023). Lin et al. further substantiated the merits of combination therapy in a murine model of PA infection. Utilizing a dry powder inhaler to administer a phage PEV20 in conjunction with ciprofloxacin, the team achieved a 5.9 log10 reduction in the pulmonary load of MDR PA (Lin et al., 2021). This approach was well-tolerated by the mice, exhibiting no adverse effects, including alveolar fibrosis or alveolar hemorrhage. These findings confirm that phage monotherapy can overcome physiological barriers in murine systems. Moreover, when combined with antibiotics, it generates synergistic bactericidal activity superior to either monotherapy, highlighting the translational promise of optimized combinatorial regimens.

Recent studies have also achieved notable breakthroughs in the field of mammalian models for MDR PA infections that are clinically refractory. According to the findings reported by Rezk et al., the application of phage ZCPA1 resulted in a wound closure rate of 99.84% (control: 69.66%) and a 4 log10 reduction in MDR PA bacterial loads in a rat model of total skin defects. This treatment also promoted epidermal and skin accessory regeneration (Rezk et al., 2022). Guillon’s team demonstrated a 1.5 log10 reduction in pulmonary bacterial load in a porcine model of mechanically ventilated pneumonia by nebulizing phage cocktails. The serum phage titers were consistently below the limit of detection (less than 10^2^ PFU/mL), confirming the local efficacy and systemic safety of the phage cocktails (Guillon et al., 2021). Collectively, these findings establish a comprehensive chain of evidence spanning from *in vitro* assays to *in vivo* animal models. This evidence offers novel approaches to address the challenges posed by MDR PA, including the biofilm barrier and multidrug resistance.

## 3 Clinical reports of phage therapy for PA infections

### 3.1 Reprogramming the treatment of artificial vessel infections

Chan et al. (2018) reported a case of paradigm-shifting treatment of aortic graft infection caused by MDR PA. In a 76-year-old male patient with recurrent postoperative artificial vascular infection due to MDR PA, researchers achieved a major clinical breakthrough in MDR PA infection by local injection of phage OMKO1. This approach not only induced a 50% reduction in ceftazidime MIC and a 90% reduction in ciprofloxacin MIC but also significantly disrupted the structural integrity of the biofilm of the drug-resistant PA. This evolutionary trade-off resulted in a synergistic bactericidal effect of the phage-antibiotic combination, which significantly reduced the bacterial load in the bloodstream. Consequently, artificial vascular infections were successfully cured after treatment, and the incidence of bacteremia and other disorders during the treatment period was drastically reduced.

### 3.2 Biofilm breakthroughs in joint infections

Recent studies have demonstrated the efficacy of phage-antibiotic combinations in addressing the therapeutic dilemma of artificial joint infections caused by MDR PA. In a knee infection model, arthroscopic administration of a trivalent phage mixture (PP1450/1777/1792) with oral ciprofloxacin resulted in a substantial decrease in the MDR PA biofilm, and the patient achieved successful clinical cure (Ferry et al., 2021). In a hip infection model, researchers used a Pa53 phage + meropenem regimen that resulted in a 4-fold decrease in drug-resistant PA minimal biofilm-removal concentration (Cesta et al., 2023). The combination of these two cases demonstrates the pivotal function of localized high-concentration delivery in the penetration of the biofilm of drug-resistant PA. Furthermore, it suggests that a therapeutic strategy involving the combination of antibiotics and phages enhances therapeutic efficacy and offers a novel approach for addressing MDR bacterial infections.

### 3.3 Precise eradication of device infections

Racenis et al. (2023) established a spatio-temporal synergistic treatment program for left ventricular assist device-associated infections. The team’s regimen of local and intravenous phage PNM and PT07 (10^7^ PFU/mL) combined with catheter reset resulted in an approximately 70% decrease in traumatic MDR PA load after 8 days of treatment. Furthermore, a significant reduction in biofilm biomass—a primary driver of drug resistance in PA—was observed. Following the conclusion of the treatment regimen, no recurrences were observed in the 21-month follow-up period. This finding suggests that patients undergoing this treatment regimen successfully attained complete clearance of drug-resistant PA, thereby validating the regimen’s capacity to maintain sterilization.

### 3.4 Integrated treatment of multisystem infections

In the management of burns secondary to ventilator-associated pneumonia with bacteremia due to MDR PA infection, researchers developed a personalized nebulized-intravenous multimodal delivery system (Teney et al., 2024). The co-treatment of phages and imipenem-rapatan led to a reduction in bronchial secretions, a decrease in oxygen dependence, and successful extubation without relapse after a treatment period of 7 days. It is noteworthy that this study also yielded a second-generation phage mixture, which could be utilized in the future to ascertain the activity of the second-generation mixture against MDR PA selected in the initial round of phage treatment. Subsequent to this determination, it is possible to ascertain whether the selection of phage-resistant mutants compromises clinical response and provides a resistance management program for sequential therapy.

## 4 Clinical trials of phage therapy for PA infections

An investigation was conducted on the early first-in-human clinical trial of a hexavalent lysogenic phage cocktail for the treatment of otitis media caused by PA infection (Wright et al., 2009). A notable decrease in PA colony-forming units was observed in the treatment group compared with the placebo group. Additionally, a mean phage residency time of 23.1 days was recorded in 24 adult patients, indicating the potential for host clearance or the evolution of PA resistance. However, no serious adverse events were reported, which initially validates the safety of topical administration.

The subsequent Phase II PhagoBurn study using a combination of 12 naturally lysogenic phage strains was the first to compare phage therapy with standard silver sulfadiazine therapy in a burn wound infection model (Jault et al., 2019). Notwithstanding the early termination of the study due to enrollment barriers (*n* = 27) and formulation stability issues, a subgroup analysis of the study demonstrated significant clinical improvement in patients with drug-resistant PA infection. The study also provided three core principles for the clinical use of phages in subsequent studies: (1) Optimize strain dosing through phage susceptibility mapping before treatment to reduce the risk of drug resistance and adverse effects. (2) Ensure that the formulation maintains biological activity *in vitro* and *in vivo*, especially in the case of multiphage co-application, and always pay attention to phage interactions. (3) Heterogeneous study cohorts covering multi-age and multiphenotype strains should be established to ensure the scientific validity and generalizability of the study.

Recently, a US multicenter trial evaluated the single-agent efficacy of intravenous lytic phage cocktail (WRAIR-PAM-CF1) in patients with cystic fibrosis (Tamma et al., 2022). Preliminary data demonstrate the efficacy of the aforementioned regimen in reducing drug-resistant PA load in the lungs, even in the absence of antibiotics. This is the first instance of lytic phages being used as a pharmaceutical agent in a clinical setting in the United States, which contributes to a better understanding of phage-independent antimicrobial effects in the human body and provides an important reference for future clinical applications.

According to the results of a recently conducted Phase I/II clinical trial, the topical formulation of phage TP-102 has demonstrated efficacy in treating diabetic foot ulcers caused by MDR PA infections. This development marks a significant breakthrough, as reported in the study’s findings (Nir-Paz et al., 2024). Among the 13 patients diagnosed with MDR PA infections, the study observed that 76.9% achieved wound closure of more than 50%, with one patient demonstrating complete epithelialization. The study’s results indicate the potential efficacy of phage therapy in managing complex wounds. However, prudent interpretation of these encouraging results remains imperative given the inherent statistical constraints associated with the investigation’s limited enrollment size (*n* = 13), which necessitates validation through larger-scale controlled studies.

**Figure 1:**
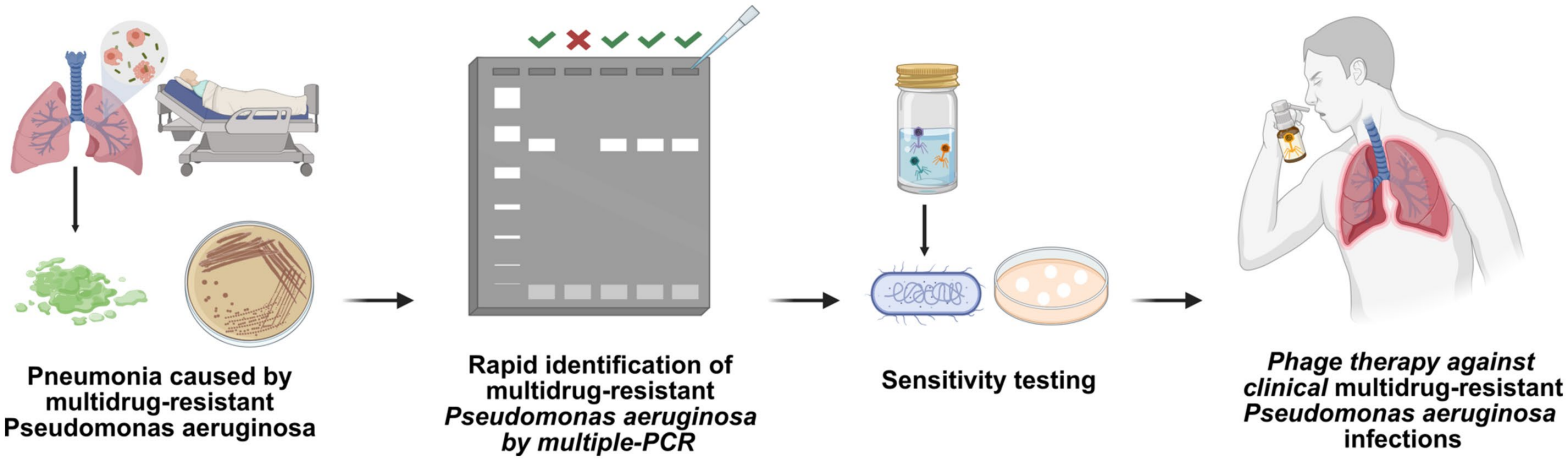
Standardized clinical protocol for phage cocktail therapy of MDR-*Pseudomonas aeruginosa* infections. The protocol involves three key steps: (1) Identification of MDR-PA isolates via multiplex PCR, (2) comprehensive phage susceptibility testing, and (3) administration of a customized phage cocktail for pneumonia treatment. Drawn by BioRender.

Collectively, clinical case reports demonstrate the potential of personalized phage therapy against MDR PA infections, while controlled trials substantiate its reproducible efficacy and acceptable safety profile. To further elucidate its clinical application, we summarize the protocol for using a phage cocktail against multidrug-resistant *Pseudomonas aeruginosa* pulmonary infections in Figure 1. To synthesize these critical clinical evidence, Table 1 systematically consolidates key therapeutic characteristics and outcomes of phage interventions for MDR PA infections, encompassing: treatment modalities, phage-host interaction dynamics, and clinical endpoints (Wright et al., 2009; Chan et al., 2018; Jault et al., 2019; Ferry et al., 2021; Tamma et al., 2022; Cesta et al., 2023; Racenis et al., 2023; Nir-Paz et al., 2024; Otava et al., 2024; Teney et al., 2024).

**Table 1:** Case reports and clinical trials.

Case reports							
Case (Reference)	Infection	Complicating conditions	Antibiotic courses	Antibiotic resistance or allergies	Phage dose and application	Duration of phage treatment	Outcome
Case 1 (Chan et al., 2018)	*Pseudomonas aeruginosa* infection of aortic graft	Aortic abscess with mediastinal fistula	Ceftazidime and Ciprofloxacin	Resistant to Ciprofloxacin	A mixture of *Pseudomonas aeruginosa* bacteriophage OMKO1 (1 × 10^7^PFU/ml) and Ceftazidime (0.2 g/mL) was administered via direct injection into the infected site.	Single dose	Resolved infection with no recurrence for 18 months post-treatment.
Case 2 (Ferry et al., 2021)	Infection of the left knee prosthesis with *Pseudomonas aeruginosa.*	Joint effusion and heart failure	Ceftazidime and Ciprofloxacin	Susceptible to ceftazidime and ciprofloxacin	A phage cocktail (1 × 10^9^ PFU/ml) was prepared and 30 mL was administered via intra-articular injection into the infected knee joint cavity	Single dose	Rapid symptom resolution of joint pain and heart failure was achieved; the left knee joint maintained full functional recovery at 1-year follow-up
Case 3 (Cesta et al., 2023)	Infection of the right hip prosthesis with *Pseudomonas aeruginosa*.	Recurrent *Pseudomonas aeruginosa* prosthetic biofilm infection (right hip)	Intravenous meropenem 2 g every 12 h	Resistant to ceftazidime and ciprofloxacin	*Pseudomonas aeruginosa*-targeted phage therapy was locally administered via surgical drainage catheter (intra-catheter instillation under sterile protocol).	Phage therapy was administered locally for 15 days, while meropenem was given intravenously for 14 days.	The patient demonstrated rapid resolution of infectious symptoms post-treatment, with sustained remission maintained throughout a 24-month follow-up period (no evidence of microbiological or clinical relapse).
Case 4 (Racenis et al., 2023)	Multidrug-resistant *Pseudomonas aeruginosa* LVAD driveline infection	Cardiomyopathy and heart failure	Ceftazidime/avibactam and amikacin	Resistant to ceftazidime and ciprofloxacin	A dual lytic bacteriophage regimen (1 × 10^7^ PFU/mL per phage) was administered through combined intravenous and topical routes.	Phage intravenous administration was continued for 8 days; daily topical application was performed postoperatively for 3 days.	Infection symptoms resolved rapidly with normalized inflammatory markers and no recurrence during 21-month follow-up.
Case 5 (Otava et al., 2024)	*Pseudomonas aeruginosa* infection complicating arterial stent placemen	Pseudoaneurysm and Neutropenia	Piperacillin/Tazobactam, Ceftazidime and Meropenem	Levofloxacin	A phage cocktail preparation (each bacteriophage component at 10^7^PFU/mL) was administered via intravenous infusion following aseptic protocol.	Bacteriophage cocktail (3-phage composition) administered via continuous IV infusion at 2 × 10^9^ PFU/day	Post-treatment, the patient’s infection biomarkers normalized, and no further *Pseudomonas aeruginosa* sepsis episodes occurred within 10 months
Case 6 (Teney et al., 2024)	*Pseudomonas aeruginosa*-induced ventilator-associated pneumonia	Bacteremia and burn infections	Ceftazidime/avibactam, Imipenem-relebactam, Inhaled polymyxin B	Carbapenems, Quinolones, Aminoglycosides	Two bacteriophages (PP1792 and PP1797) were administered at concentrations of 2 × 10^9^PFU/mL (inhalation) and 2 × 10^8^PFU/mL (intravenous injection), respectively	The treatment regimen consisted of 7-day bacteriophage therapy combined with 14-day antibiotic therapy using imipenem-relebactam and inhaled polymyxin	The treatment rapidly improved ventilator-associated pneumonia and bacteremia, with no severe *P. aeruginosa* recurrence within 1 month

Clinical trials						
Case (Reference)	Infection	Trial	Treatment group	Placebo group	Phage dose and application	Outcome
Case 1 (Wright et al., 2009)	Chronic otitis media caused by *Pseudomonas aeruginosa*	A randomized, double-blind, placebo-controlled phase I/II clinical trial	Twelve subjects received a bacteriophage cocktail	Twelve subjects received glycerol-PBS buffer solution.	A single intra-aural administration of a six-phage cocktail	The treatment group showed significant clinical improvement at 7,21and 42 days post-treatment, whereas no significant improvement was observed in the placebo group
Case 2 (Jault et al., 2019)	Burn wound infection primarily caused by *Pseudomonas aeruginosa*	A randomized, double-blind, placebo-controlled phase I/II clinical trial	Topical application of a bacteriophage cocktail	Topical application of 1% silver sulfadiazine cream	Alginate dressing soaked in diluted bacteriophage cocktail (10^9^ PFU/mL) applied directly to wound	The phage-treated group showed slower bacterial load reduction by treatment endpoint (Day 7), though partial clinical improvement was still observed in some patients
Case 3 (Tamma et al., 2022)	*Pseudomonas aeruginosa* infection in the airways of cystic fibrosis patients	Multicenter, randomized, double-blind, placebo-controlled trial	Single-dose intravenous administration of a bacteriophage cocktail	Single-dose intravenous placebo administration	The starting dose was 4 × 10^7^ PFU, with each cohort receiving a 10-fold dose escalation. The bacteriophage cocktail was diluted in normal saline to the target concentration prior to intravenous administration	Interim analysis demonstrated reduced *Pseudomonas aeruginosa* colony counts in the bacteriophage-treated group, with no severe adverse events observed
Case 4 (Nir-Paz et al., 2024)	*Pseudomonas aeruginosa* infection in diabetic foot ulcers (DFU)	Randomized, double-blind, placebo-controlled trial	Topical administration of bacteriophage cocktail TP-102	Topical administration of placebo	The bacteriophage cocktail at a concentration of 10^9^ PFU/mL/cm^3^ is directly administered to the targeted ulcer for localized treatment	No treatment-related serious adverse events (SAEs) were reported in the treatment group. The treatment demonstrated excellent tolerability and a favorable safety profile in patients with diabetic foot ulcers

## 5 New technologies for phage therapy overcoming natural phage stability deficiencies

**Figure 2:**
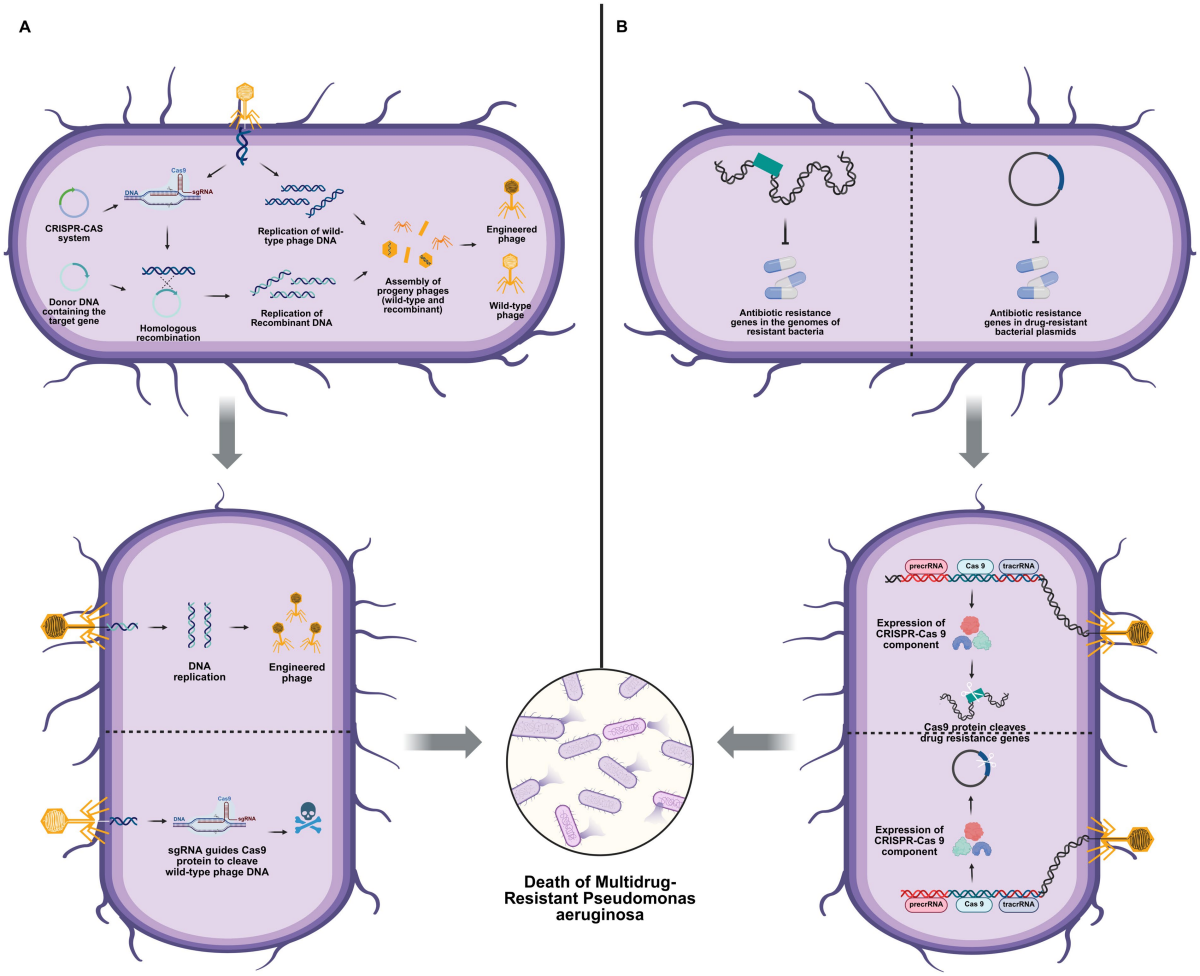
CRISPR-Cas systems for phage engineering and combating antibiotic-resistant bacteria. **(A)** Recombinant engineering enhanced by CRISPR counterselection. Donor DNA containing the target gene is incorporated into the phage genome via homologous recombination. The CRISPR-Cas system, guided by sgRNA, selectively cleaves wild-type phage DNA, thereby enriching for recombinant phage progeny that carry the desired genetic modification. This counterselection strategy enhances the efficiency of recombinant phage production. **(B)** Combating drug-resistant bacteria using the CRISPR-Cas system. The CRISPR-Cas9 system is expressed within the bacterial cell and programmed to cleave antibiotic resistance genes located either in the bacterial chromosome or on plasmids. This targeted DNA disruption resensitizes bacteria to antibiotics, prevents the spread of resistance, and ultimately leads to the eradication of drug-resistant bacteria. Drawn by BioRender.

Despite the efficacy of phage therapy in eradicating MDR PA, challenges such as instability persist. The recent advancements in phage genome engineering, a novel technology, have presented a novel solution to address the stability limitations of natural phages and expand their therapeutic range. Through the precise modification of phage genetic elements, researchers can effectively regulate their biological behaviors. For instance, deletion of the lysogeny maintenance gene *pflM*, has been demonstrated to be a successful method for achieving this objective (Schmidt et al., 2024). The development of phage genome engineering also includes the construction of shuttle phages (Pang et al., 2022), direct cloning (Bomfiglio et al., 2025), reorganization projects (Zheng et al., 2023), CRISPR-Cas selection (Cheng et al., 2020), and recombinant engineering combined with CRISPR-mediated counterselection (Mahler et al., 2023). In recent years, the fusion technology of the CRISPR-Cas system and recombination engineering techniques has enabled the rapid and precise regulation of phage genomes; the shuttle phage platform based on the *λ* red recombination system enables efficient insertion of >30 kb of exogenous DNA, while the CRISPR-mediated counter-selection strategy shortens the iterative optimization cycle of targeted genes to 48 h (Leal et al., 2024). A recent study by Qin’s team, employing PA as a model, has substantiated the clinical translational potential of these technologies (Qin et al., 2022). The construction of broad-spectrum engineered phage EATPs was successfully achieved by introducing a Type I anti-CRISPR (AcrIF1, AcrIF2, and AcrIF3) genes into the phage DMS3 genome. The DMS3*_acrIF1_* exhibited complete inhibition of 27 clinical isolates when potency reached 1 × 10^8^ PFU/mL (MOI = 0.2; wild-type phage 60–80% inhibition). The mechanism of action of DMS3*_acrIF1_* may involve specific interference with the host CRISPR-Cas adaptive immune pathway. This study provides foundational evidence that engineered phages constitute a promising and versatile platform for antimicrobial therapy. The general principles underlying this engineered phage platform and its application for the targeted elimination of antimicrobial resistance are summarized in Figure 2. The successful application of phage genome engineering not only validates its considerable potential for clinical translation but also significantly broadens the therapeutic applicability of phage-based strategies against challenging MDR PA infections.

Furthermore, the application of genome sequencing technology can enhance the safety and efficacy of engineered phage therapies against MDR PA infections. Sequence conservation of the PA terminase large subunit can be resolved to model the risk assessment of phage-mediated horizontal gene transfer (Niazi et al., 2020; Lokareddy et al., 2022). Concurrently, macro genomic association analysis enables systematic screening of PA-associated virulence elements (Cortes-Lara et al., 2021; Valiatti et al., 2024). Therefore, this technique can be applied in clinical MDR PA strains to detect risk factors such as cryptic plasmids and virulence islands. Notably, the evolutionary rate of PA phage receptor genes was significantly higher than that of housekeeping genes, an observation that provides a molecular marker for predicting the evolution of PA resistance (Marchi et al., 2023). Despite the advancements in technology, which have transitioned from single-gene editing to multi-module synergistic regulation, the long-term ecological effects of engineered phages require meticulous evaluation. Future advancement mandates establishing global phage genome databases to enable real-time virulence tracking, dynamic monitoring of host-phage coevolutionary networks, and continuous therapeutic optimization through adaptive surveillance.

## 6 Effect of immune response on phage treatment of PA infection

**Figure 3:**
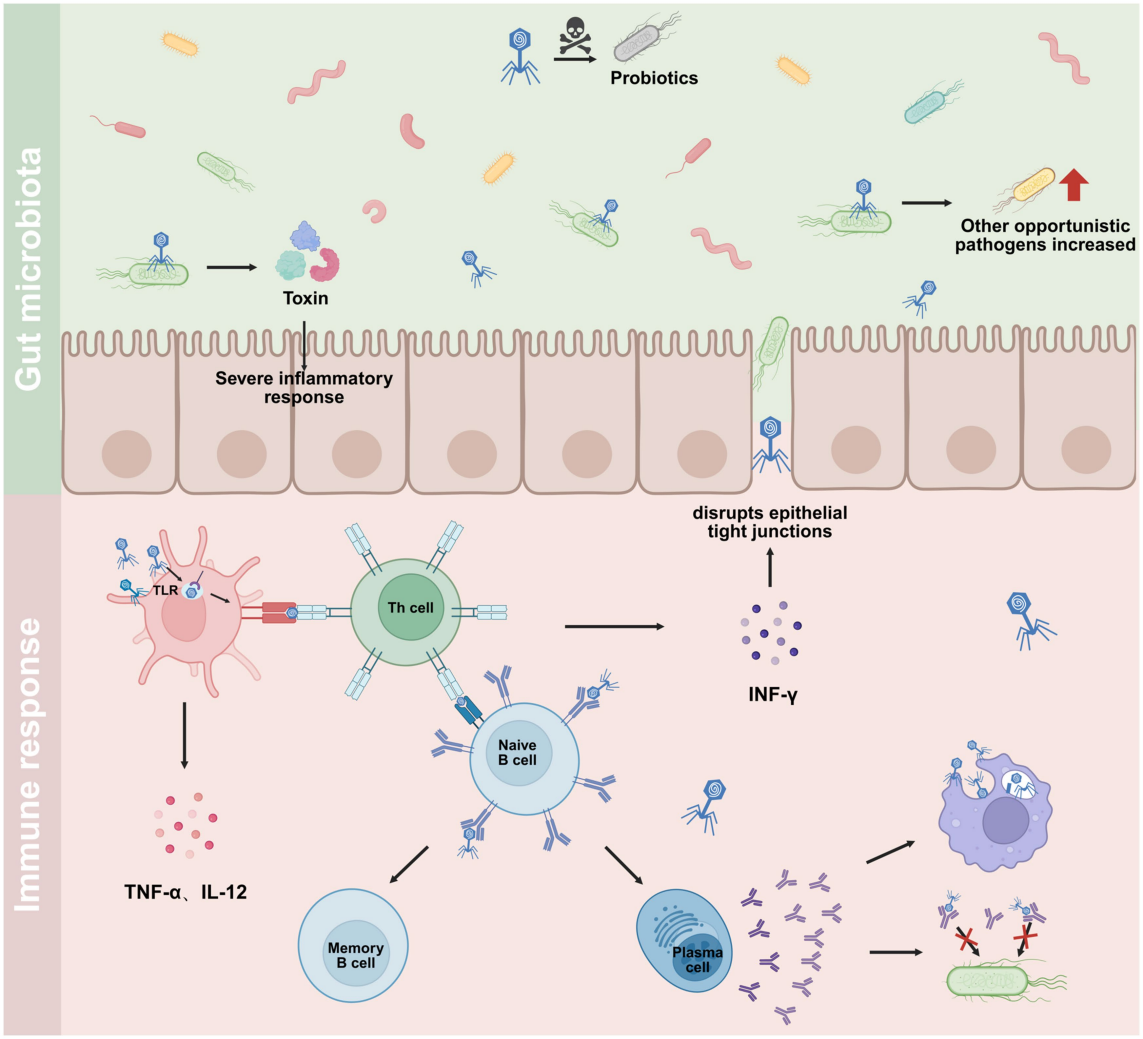
Biosafety considerations and immune responses in phage therapy. Upper panel: Ecological and inflammatory risks. (1) The lysis of bacterial cells releases pathogen-associated molecular patterns, which can trigger a severe inflammatory response through host pattern recognition receptors. (2) Phages may lack absolute specificity and could potentially target beneficial commensal bacteria that share epitopes with the intended pathogen. (3) The successful elimination of a target pathogen (e.g., *Pseudomonas aeruginosa*) creates an ecological niche, potentially facilitating the overgrowth of other opportunistic pathogens and leading to new infections. Lower panel: Antiphage immune activation. Phages themselves can elicit both innate and adaptive immune responses. This includes the induction of pro-inflammatory cytokines (e.g., TNF-*α*, IL-12, IFN-*γ*), activation of T-helper cells, and subsequent stimulation of B cell differentiation into antibody-producing plasma cells and memory B cells. The resulting phage-specific antibodies can neutralize the antibacterial activity of phages, reducing the active phage titer. Furthermore, these antibodies also promote the rapid clearance of phages from the body by facilitating their uptake and degradation via phagocytosis. Drawn by BioRender.

As a multiprotein complex, phages possess significant immunogenicity due to their structural components, including coat proteins and tail filament proteins. These components have the capacity to elicit specific humoral and cellular immune responses (Figure 3). As indicated by previous studies, a significant number of phage structural proteins have been shown to play an antigenic role in the humoral immune response against phages after entering the human body (Gembara and Dąbrowska, 2021; Dan et al., 2022; Zou et al., 2023; Podlacha et al., 2024). Moreover, anti-phage IgG levels in the sera of PA patients after intravenous phage injection were 100-fold higher than those in the intravenous immunoglobulin group and 1,000-fold higher than those in the normal, healthy donor group (Dan et al., 2022). Mechanistic studies demonstrated that CD4^+^ T cell subsets, particularly activated circulating T follicular helper cells, reached their peak on Day 14 after administration. This was accompanied by a low expression of Th1-type cytokines (e.g., IFN-*γ*, IL-2) and a significant elevation of pro-inflammatory factors, such as IL-6 and GM-CSF. These findings suggest that phages may activate intrinsic immunity through relevant pathways. A combined analysis of the study indicated that phage therapy led to a substantial enhancement in immune responses among patients afflicted with MDR PA infections, signifying a favorable development in the management of such infections. However, this robust immune activation may also incur potential risks, including excessive inflammatory responses or autoimmune reactions. Consequently, close monitoring of the patient’s immune status is imperative in clinical applications to ensure the treatment’s safety. It is worth noting that the intensity of the immune response is strongly correlated with the administration route. Specifically, intravenous and peritoneal injections have been shown to elicit a higher antibody neutralization rate compared to oral administration (de Kretser et al., 2024). This phenomenon may be attributed to differences in antigen presentation thresholds for mucosal immunity.

During phage therapy for MDR PA infection, some neutralization occurs between phages and phage-specific antibodies (Figure 3), exhibiting high heterogeneity. A controlled study by Dedrick et al. demonstrated that elderly patients with bronchiectasis due to MDR PA infection treated with the same phage cocktail exhibited a significantly higher clinical failure rate than the younger group, attributable to the neutralization of phages by preexisting IgG (Dedrick et al., 2019). This finding suggests the potential for variability in the neutralization of the phages in question among different age groups of humans, necessitating further research to ascertain the specific impact of this variability on therapeutic efficacy. However, although phage therapy has been demonstrated to induce a variety of phage-specific antibodies, not all antibodies exhibit phage-neutralizing activity. For example, in a recent phage therapy report, PA head protein-specific IgG titers were not statistically different between serum neutralization-positive and negative groups, suggesting that epitope specificity, rather than total antibody volume, determines neutralization efficacy (Washizaki et al., 2024). The intricacies of phage neutralization underscore the necessity to devise a phage immune cross-reactivity map for MDR PA. This endeavor necessitates a systematic resolution of the conserved B-cell epitopes present within the diverse phage coat proteins, in addition to the establishment of a comprehensive database encompassing neutralizing antibody epitopes.

## 7 Biological safety evaluation of phage therapy

Despite the significant therapeutic potential of phage therapy, its biosafety profile requires systematic evaluation prior to clinical translation. Preclinical and clinical studies provide multidimensional evidence supporting its general safety. As described in Section 2.2, animal models, including zebrafish, rodents, and pigs, have preliminarily validated its *in vivo* tolerability. For instance, the PEV20 phage combined with ciprofloxacin in a dry powder inhaler, as used by the Lin team, did not induce pathological damage such as pulmonary fibrosis or hemorrhage in mice (Lin et al., 2021). Guillon *et al.* demonstrated in mechanically ventilated pneumonia pig models that serum titers of nebulized phage cocktails remained persistently below detection limits (<10^2^ PFU/mL), indicating localized pulmonary action with low systemic diffusion risk (Guillon et al., 2021). Clinical evidence from subsequent studies further corroborates this safety profile (Sections 3 and 4). The PhagoBurn trial and clinical trials in cystic fibrosis patients in the United States, further supported the safety of phage administration via both topical and intravenous routes (Jault et al., 2019; Tamma et al., 2022). Notably, in the TP-102 trial for diabetic foot ulcers, all adverse events were assessed as unrelated to phage therapy (Nir-Paz et al., 2024). These findings collectively demonstrate that rigorously prepared phage formulations exhibit a favorable safety and tolerability profile in humans.

A core biosafety dilemma, however, revolves around the host immune response to phages as exogenous antigens. While moderate immune activation may aid infection clearance, the production of neutralizing antibodies poses a critical risk to therapeutic efficacy (Majewska et al., 2019; Dhungana et al., 2021; Herren et al., 2024; Vila et al., 2024). This dynamic was evident in a case reported by Bernabéu-Gimeno et al. (2024) where antibody-mediated pressure can diminish efficacy over time, particularly in chronic infections like cystic fibrosis, and may even select for immune-evading phage mutants. This underscores a complex co-evolutionary process involving phages, host immunity, and bacteria. Beyond immunity, the ecological impact on human microbiomes warrants attention. Although highly specific, phages could theoretically infect non-target commensals phylogenetically close to the pathogen, potentially disturbing microbiota with unknown long-term consequences. This potential for non-target effects and ecological disruption is illustrated in Figure 3.

To address these challenges and advance phage therapy from experimental to routine application, the establishment of a comprehensive regulatory and monitoring framework is imperative. This necessitates: prior to administration, rigorous risk assessment must be conducted, including endotoxin testing of formulations, whole-genome sequencing to exclude virulence, and precise determination of host range; during treatment, real-time dynamic monitoring should be implemented using standardized pharmacokinetic and immunodynamic protocols to track phage kinetics, neutralizing antibody titers, and inflammatory markers; and following treatment, long-term follow-up is essential to monitor for infection recurrence, restoration of the microbiome, and any delayed immune responses. Such a structured approach is crucial for ensuring biosafety and building the evidence base required for widespread clinical adoption.

## 8 Advantages and challenges of phage therapy

Phage therapy has been demonstrated to exhibit several advantages over antibiotic therapy. First, phages are distributed extensively and in high numbers across various ecological niches, enabling expeditious isolation. Consequently, the financial burden associated with phage development is considerably less substantial in comparison to that of antibiotics, which are often challenging to acquire (Liu et al., 2021; Nair et al., 2022). Second, unlike antibiotics that persist systemically and provoke off-target damage to commensal microbiota (frequently antibiotic-associated diarrhea), phages self-limit through natural depletion post-therapy. This intrinsic clearance mechanism eliminates treatment-related dysbiosis risks, ensuring exceptional safety throughout clinical administration (Lin et al., 2022; Vaezi et al., 2024). Third, phage therapy exerts a substantial anti-inflammatory effect, as demonstrated by a reduction in C-reactive protein levels and a decrease in white blood cells (Farfán et al., 2022; Cao et al., 2025). Fourth, phages have significant host specificity, ensuring that non-host bacteria are largely immune to phage attacks (Luong et al., 2020). Hence, phages do not interfere with the normal metabolism of human cells. Finally, the rate at which phage resistance develops is 10 times slower compared to the rate at which antibiotic resistance develops (Kaneko et al., 2023). This means that there is relatively greater potential to utilize phage therapy in the face of bacterial resistance, as bacterial resistance to phage develops more slowly, contributing to improved therapeutic efficacy and prolonged treatment duration.

Phages offer unique therapeutic advantages but still face numerous challenges. These challenges include the need for more efficient phage screening methods, as current approaches are often labor-intensive and time-consuming; the typically narrow host range of individual phages, which limits their applicability against diverse bacterial strains; and the difficulty in developing effective treatment strategies for infectious biofilms, which are structured bacterial communities inherently resistant to penetration. Moreover, scaling up phage production faces hurdles in maintaining batch-to-batch consistency. Significant standardization challenges also remain, particularly regarding two critical aspects: phage stability (as they are sensitive to temperature and pH fluctuations) and purity assurance (Plumet et al., 2022). The current clinical evidence on optimal phage dosing or PK parameters for treatment is quite limited, precluding the accurate derivation of a specific dosing regimen for a given phage (Chang et al., 2022). Consequently, there is a compelling need for further research on the inhibitory effects of phage therapy on MDR PA in clinical and *in vitro* studies to facilitate the broader implementation of this therapeutic approach.

## 9 Conclusion

In this review, we provided a synopsis of recent advancements in phage therapy for the treatment of MDR PA. The global epidemic of MDR PA has constituted a major public health crisis, compelling the medical community to revisit the path of revolutionizing antimicrobial strategies. Phages demonstrate distinct advantages in their multifaceted synergistic interactions with antibiotic-resistant bacteria. Their capacity to target and eliminate bacteria with high specificity renders them a promising alternative to conventional antibiotics. Phages offer several advantages, including enhanced safety, the potential to restore bacterial susceptibility to antibiotics, and significant biofilm disruption. However, there is a paucity of knowledge regarding the application of phage therapy. The clinical application of phages is currently limited by the paucity of human studies, and the laboratory practices currently in place do not support their use. The temporal and mechanistic intricacies of antibiotic administration in conjunction with phage therapy remain to be elucidated, particularly concerning its influence on the efficacy of antibiotic combination therapy. Consequently, this juncture presents a propitious opportunity for basic researchers and physicians. Looking forward, elucidating phage–antibiotic synergies and developing engineered phage cocktails may pave the way for novel combination regimens capable of overcoming existing resistance mechanisms. These developments are expected to bridge fundamental research and clinical practice, offering new frameworks for antimicrobial drug development. The implementation of appropriate regulatory measures and the optimization of intellectual property policies are expected to result in a more significant impact of phages on the life sciences field. As awareness of phage therapeutics continues to grow, their clinical applications and related industries are expected to see significant growth in the near future.
